# Income Level but Not Nutrition Knowledge Is Associated with Dietary Diversity of Rural Pregnant Women from Northern Ghana

**DOI:** 10.1155/2021/5581445

**Published:** 2021-07-12

**Authors:** Emmanuel Amoako Agyei, Stephen Kofi Afrifa, Adam Munkaila, Patience Kanyiri Gaa, Eugene Dogkotenge Kuugbee, Victor Mogre

**Affiliations:** ^1^Department of Community Health and Family Medicine, School of Medicine and Health Sciences, University for Development Studies, Tamale, Ghana; ^2^Department of Obstetrics and Gynaecology, School of Medicine and Health Sciences, University for Development Studies, Tamale, Ghana; ^3^Nutrition Unit, Tamale Central Hospital, Ghana Health Service, Tamale, Ghana; ^4^Department of Clinical Microbiology, School of Medicine and Health Sciences, University for Development Studies, Tamale, Ghana; ^5^Department of Health Professions Education and Innovative Learning, School of Medicine and Health Sciences, University for Development Studies, Tamale, Ghana

## Abstract

Essential nutrients are necessary for reducing the risk of maternal mortality, prenatal mortality, and low-birthweight infants. Dietary diversity can play an important role in supplying essential nutrients to both the mother and the foetus. We evaluated nutrition knowledge, attitudes, and dietary diversity of pregnant women. In addition, we investigated the sociodemographic determinants of dietary diversity among pregnant women from a rural district in Ghana. Participants were pregnant women receiving antenatal care from a rural district hospital in Ghana. Dietary diversity was measured using a 24-hour dietary recall questionnaire. Multiple linear regression was used to determine the sociodemographic characteristics of dietary diversity. About 85% of the pregnant women knew that they should eat more in comparison to nonpregnant women, and only 16.9% knew the importance of folic acid supplementation during pregnancy. Mean (SD) dietary diversity score of the participants was 5.27 (1.35), 85.4% did not consume any fruits, and 82.3% did not take milk and milk products. Almost all participants took at least one food item in the starchy staples and green leafy vegetables food groups. Moreover, 53% consumed vitamin A-rich fruits, vegetables, and tubers; 7.7% organ meats; and 30.8% eggs. Those who earned a monthly income of ≥GHC 500 or US$ 87 (*B* = 1.82; 0.90–2.73; *p* < 0.001) significantly had higher dietary diversity scores compared to those who earned less. Dietary diversity of the pregnant women was suboptimal. The consumption of vitamin A- and iron-rich foods was inadequate. Income was an important determinant of the dietary diversity of pregnant women from Northern rural Ghana.

## 1. Introduction

Nutrition during pregnancy is a basic determinant of foetal growth, birthweight, and infant morbidity as poor nutrition often leads to long-term, irreversible, and detrimental consequences to the foetus [1]. Evidently, various studies show that inadequate intake of energy or particular nutrients during pregnancy can have a negative impact on the health of the newborn later in life [2]. Malnutrition in infancy and childhood is greatly influenced by foetal malnutrition which may result in intrauterine growth restriction (IUGR) [1].

Pregnancy is a critical period that requires the intake of varied and diverse diets in order to meet the high nutrient needs of the developing foetus and the mother. Dietary diversity is one of the ways by which individuals including pregnant women could improve their dietary intake. It is frequently reported as a measure of diet quality and refers to the number of foods consumed across and within food groups usually over the last 24 hours [3–5]. It follows the concept that adequate intake of essential nutrients is assured by increasing the diversity of foods and food groups in the diet. Poor dietary diversity may result in inadequate intake of nutrients which is reportedly common among pregnant women from sub-Saharan Africa [6, 7].

A number of factors have been previously reported for this situation including cultural practices, food availability, unaffordability, poor income, seasonality, appetite, taste, nausea, and misconceptions [6–8]. In low-income countries, it is particularly challenging for women to meet macro- and micronutrients requirements during pregnancy. This challenge is in part due to inadequate nutritional competence (knowledge, attitude, and practice), as evidenced by a previous study stating that 72% of pregnant women chose the kinds of foods they ate based on food beliefs and what they hear from influential people like their grandparents [9]. A study in Northern Ghana showed that 68.3% of pregnant women had poor dietary knowledge and 31.7% had good dietary knowledge [10]. In a study among pregnant women in Yaounde, Cameroon, participants had sufficient knowledge of the appropriate dietary requirements in pregnancy but did not literally translate it into optimal dietary practice [11]. The authors further reported that sociocultural beliefs and food taboos prevented participants from consuming foods such as meat obtained from nondomestic animals, chicken, and sea fish and thus had a significant influence on maternal dietary habits [11]. Another study among pregnant women from Kenya reported dietary diversity scores of 6.84 (1.46), and the most eaten food group was cereals [12]. Adequate maternal nutrition knowledge and dietary practices before and during pregnancy are necessary to ensure positive pregnancy outcomes [11]. A study from Ghana reported that poor dietary knowledge of pregnant women affected the health of pregnant women and babies leading to conditions such as anaemia in pregnancy and malnutrition in babies [13].

The evaluation of dietary diversity among pregnant women may offer an invaluable foundation in quest of the enhancement of nutrition of the pregnant woman and her unborn child. However, evidence on dietary diversity of pregnant women and its determinants is limited. In our review of the literature, we came across only three studies from Ghana [10, 14, 15] and a few studies from other parts of sub-Saharan Africa [11, 12, 16–21] investigating the phenomenon. One of the studies from Ghana followed a qualitative approach [15], and although the other two studies used a quantitative approach [14], one of them did not investigate the nutrition-related knowledge and attitudes of pregnant women and how these may affect the dietary practice of pregnant women, and the other study investigated these but among pregnant women in Accra, which is economically different from the setting of the current study. This study investigated nutrition-related knowledge, attitudes towards nutrition, and the socio-demographic determinants of dietary diversity among pregnant women in rural Ghana.

## 2. Materials and Methods

### 2.1. Study Design and Setting

This cross-sectional study was carried out in Gwollu District Hospital in the Sissala West district. The Sissala West district is located in the northeastern part of Ghana. It shares boundaries with Lambussie district to the west, Sissala East district to the east, Burkina Faso to the north, and Wa East district to the south. The settlement is mainly rural and has a sparse and scattered population with a population density of 8 persons per square kilometer, occupying a total land size of 411,289 km, which is about 25% of the land mass of the Upper West Region, and road network is about 3,309.62 km with a length of 281.15 km. The district has five subdistricts with six health centers, one hospital, and 29 functional CHPS zones. These facilities offer various levels of public health and clinical services.

### 2.2. Study Participants and Sample Size Determination

Participants of this study were pregnant women attending the antenatal clinic at the Gwollu District Hospital. The antenatal clinic runs a daily antenatal clinic for pregnant women scheduled to visit monthly to receive antenatal care including nutrition and diet counseling and to receive recommended dietary supplements such as folic acid and iron. Participants were eligible to participate if they were registered at the clinic and had visited at least once. Those who were acutely ill were not eligible to participate in the study. The sample size was determined using the Yamane's formula, *n* = *N*/[1 + Ne^2^], where *n* is the sample size, *N* is the population size (183, i.e., the average ANC attendance per month at the Gwollu District Hospital for the year 2019), and *e* is the acceptable sampling error which is 5% (0.05). These were computed by the formula to yield *n* = 183/{1 + 0.05^2^ (183)} = 125. An error margin of 5% was allowed for nonresponses to yield a total sample size of 130.

### 2.3. Recruitment and Data Collection Procedures

Permission was obtained from the medical director of the hospital and the head of the antenatal clinic to obtain access to the premises to meet the study participants. SKA and EAA visited the antenatal clinic daily during the study period (i.e., February to March 2020) to recruit participants. Potential participants were approached after they had received their antenatal care. The study was introduced to them, and those who were willing to partake were taken through the consent procedures. Only those who consented were recruited into the study and given a questionnaire. The questionnaire was self-administered for those who could read and write in English. For those who needed assistance in English, two translators administered the questionnaire to them in the local dialect. All data was collected in a comfortable secluded room at the hospital.

### 2.4. Ethics Statement

The study as well as all data collection and consent procedures was approved by the Tamale Teaching Hospital Ethical Review Committee. Written informed consent was obtained, and participation in the study was voluntary. Participants were at liberty to withdraw from the study at any time if they felt uncomfortable. Confidentiality of the data was assured.

### 2.5. Data Collection Methods

Data was obtained with the use of a structured questionnaire. Participant's nutrition-related knowledge, attitudes, and practices were assessed using the Food and Agriculture Organisation (FAO) guidelines for assessing nutrition-related knowledge, attitudes, and practices [4]. The nutrition knowledge scale consisted of 5 items that assessed nutrition during pregnancy and breastfeeding, micronutrient supplements (including folic acid and iron) for pregnancy, health risks for low-birthweight babies, and family planning. Participants who correctly knew the answers to the questions were rated “know,” and those who answered incorrectly were rated “do not know.” For each of the questions, a correct answer was scored 1 and an incorrect answer scored 0. A total score was generated for each of the participants by summing the number of correct responses.

The nutrition-related attitude scale assessed perceived susceptibility to low birthweight, perceived severity of low birthweight, how good it was to eat more during pregnancy, and their perception of barriers or difficulties in eating more during pregnancy. The scale had 4 items, and they consisted of statements that were responded to using a 3-point Likert scale (1: “not likely/serious/good/difficult,” 2: “you are not sure/so-so,” 3: “likely/serious/good/difficult”). For each item, the response score was reflected in the result value.

Dietary diversity was evaluated using a food check list adapted from the FAO guidelines for measuring individual and household dietary diversity [3]. The food items were adapted to meet the local context of the study setting by replacing and adding locally available substitutes. Participants were asked to indicate foods they had eaten in the last 24 hours from among the list. The food items were categorized into 10 food groups: starchy staples; dark green leafy vegetables; vitamin A-rich fruits, vegetables, and tubers; other fruits and vegetables; organ meats (iron rich); eggs; meat and fish; legumes, nuts, and seeds; milk and milk products; and oils and fats. Participants' reported consumption of foods in each of the food groups was used to compute the women dietary diversity score (WDDS) in accordance with the recommendations of the FAO guidelines for measuring individual and household dietary diversity [22]. The WDDS was obtained by summing the number of unique food groups consumed in the last 24 hours [22] with the aim of being reflective of nutrient adequacy [3, 22, 23]. For instance, if a participant reported eating at least one of the foods listed in a particular group, the participant was scored 1 for that food group. A pregnant woman who consumed at least one food item from five or more food groups of the 10 food groups was considered to have met the minimum dietary diversity [22]. The questionnaire also collected information relating to the sociodemographic characteristics of the participants, including age, marital status, occupation, educational status, household size, and monthly income. The questionnaire was used to also obtain obstetric information such as gestational age, number of antenatal care visits, and parity. To ensure comprehensibility, the questionnaire was pretested among 20 participants selected from a nonparticipating community within the area having similar characteristics. This allowed for further clarification and modification of some of the items of the questionnaire. In addition, the questionnaire was evaluated for content validity by a team of nutritionists, behavioral scientists, and public health specialists. Suggested revisions were made before the study commenced.

### 2.6. Statistical Analysis

Data collected were keyed into Microsoft Excel and then transferred to the Statistical Package for Social Science (SPSS) statistics software for analysis. Descriptive statistics of frequencies, mean, and standard deviation were employed to describe the data. Marital status was categorized into married (including married, cohabiting, and living together) and not married (including single, divorced, and widowed); occupation status into employed and unemployed; educational status into high (including senior high school and tertiary level of education and low (including no formal education, primary and junior high school level); and monthly income into ≥GHC 500 and <GHC 500. WDDS was categorized into low dietary diversity (≤3 food groups), medium dietary diversity (4 and 5 food groups), and high dietary diversity (≥6 food groups) according to the FAO classifications [3]. To determine univariate factors of WDDS, Pearson correlation analysis was used for continuous variables (i.e., nutrition knowledge, attitudes, WDDS, age, number of antenatal care visits, gestation, parity, and household size), and the Student's *t*-test and one-way ANOVA were also employed for categorical variables (marital status, occupation, monthly income, and educational status) and WDDS. Multiple linear regression analysis was used to determine factors associated with the dietary diversity of the pregnant women. The independent variables included nutrition knowledge, attitudes towards nutrition, age, marital status, educational status, monthly income, occupation, household size, parity, gestation, and number of antenatal care visits. A *p* value of <0.05 was considered significant.

## 3. Results

All questionnaires were returned and found to be complete resulting in a 100% response rate. The sociodemographic characteristics of the participants are presented in Table 1. The pregnant women had a mean (SD) age of 27.3 (6.7) years; among them, 93.2% (*n* = 122) were married, 24.6% (*n* = 88) had a high level of education, and 81.5% (*n* = 106) followed the Islamic Religion.

### 3.1. Nutrition-Related Knowledge and Attitudes towards Nutrition during Pregnancy

Concerning dietary knowledge during pregnancy, 84.6% (*n* = 110) of the pregnant women knew that they should eat more in comparison to nonpregnant women, 56.2% (*n* = 73) knew the recommended supplements during pregnancy, and only 16.9% knew the importance of folic acid supplementation during pregnancy. Eighty percent of the pregnant women knew the health risk of low birthweight (LBW), and 27.7% had knowledge of the benefits of birth spacing. The mean ± SD knowledge score of the participants was 2.65 ± 1.461 out of a total score of 5. Regarding participants' attitudes towards nutrition during pregnancy, 60.8% perceived that they were susceptible to having low birthweight, 47.7% perceived low birthweight to be serious, 83.1% perceived that it was good to eat more food during pregnancy, and 33.8% perceived barriers to eating more food during pregnancy. The participants had a mean ± SD attitude score of 8.88 ± 1.36.

### 3.2. Women Dietary Diversity

Table 2 shows the proportion of pregnant women who took one or more foods from the various food groups. More than three-quarters (85.4%, *n* = 111) of the participants did not consume any fruits, 82.3% did not take milk and milk products, and almost all pregnant women took dark green vegetables. The mean ± SD women dietary diversity scores (WDDS) of the pregnant women was 5.27 ± 1.35. In all, 74.6 (*n* = 97) of the pregnant women met the minimum dietary diversity.

Almost all (97.7%, *n* = 127) women reportedly said there were no food taboos for pregnant women.

### 3.3. Factors Associated with Women Dietary Diversity Scores

Student's *t*-test and Pearson correlation were used to determine univariate factors of dietary diversity as presented in Tables 3 and 4. Those with a high level of education and those who earned ≥GHC 500 had higher mean WDDS compared to their counterparts.

Antenatal visits (*r* = 0.304; *p* < 0.001) and nutrition knowledge (*r* = 0.395; *p* < 0.001) correlated positively with WDDS (Table 4).

In a multiple linear regression analysis, those who earned ≥GHC 500 (US$86) (*B* = 1.82; 0.90–2.73; *p* < 0.001) remained significantly associated with higher WDDS (Table 5).

## 4. Discussion

In this study, we evaluated nutrition knowledge, attitudes towards nutrition during pregnancy, practices (women dietary diversity), and their associated factors among pregnant women from a rural district in Ghana.

Almost 40% of the pregnant women did not perceive that they were at risk of having low-birth weight babies, and more than half of them did not perceive that low-birth weight was serious. This is concerning given that a good proportion of the women may not take the needed measures to eat well to prevent LBW. Going by the Health Belief Model [24, 25], the adoption of healthy behaviors is determined by the individual perception of the severity of the condition, and if the pregnant women do not perceive that they are at risk of giving birth to low-birthweight babies, they may not adopt healthy dietary habits. It is recommended that health professionals create awareness among pregnant women regarding the risk and dangers of low birthweight during routine antenatal care.

Although over 80% of the pregnant women perceived that it was good to eat more food during pregnancy, more than 30% perceived difficulty in doing so during pregnancy. Barriers to dietary diversity during pregnancy have been reported previously by studies from sub-Saharan Africa and other parts of the world. Some of these factors include unaffordability of foods, unavailability of desired foods, pregnant women's misperception of restricted foods during pregnancy to have smaller babies, cultural practices, and taboos [15, 26]. Healthcare professionals should support pregnant women to overcome some of these barriers by encouraging them and providing them with appropriate recommendations through individualized counseling as this will afford healthcare providers an understanding of the specific needs and barriers of each pregnant woman.

Almost all pregnant women took at least one food item in the starchy staples and green leafy vegetables food groups. This is similar to a study involving pregnant women in the Greater Accra region of Ghana where a majority (99.1%) of the participants consumed starchy staples [10]. The findings also parallel those of Saaka et al. [14] among pregnant women living in rural districts of Northern Ghana in which 99.3% consumed foods from the starchy staple food group. These findings are consistent with the diets of the general population as most Ghanaian diets are predominantly starchy staples.

Another important finding of this study was that the consumption of vitamin A-rich foods was suboptimal or less encouraging given that only 53% of the pregnant women consumed vitamin A-rich fruits, vegetables, and tubers. Worryingly, the consumption of animal sources of vitamin A-rich foods was even much lower and inadequate given that only 7.7% of the pregnant women consumed organ meats, 30.8% eggs, and 7.7% milk and milk products. These findings are not peculiar to this study as Saaka et al. [14] have reported similar findings among pregnant women from Northern Ghana. These findings concern the important role of vitamin A in pregnancy as its deficiency may cause preterm birth and infant mortality [27]. Although dark green leafy vegetables were consumed by almost 94% of the participants which may compensate for the poor consumption of the animal sources of vitamin A among the pregnant women, this may not be adequate given that plant sources of vitamin A are not as active and readily available for absorption as animal sources and are also prone to poor cooking practices such as overheating that may denature the vitamin. It is therefore important for healthcare professionals to encourage pregnant women to consume animal sources of vitamin A.

Similar to vitamin A, we found that the consumption of animal sources of iron such as organ meat, flesh meat, and fish was low and less encouraging. Animal sources of iron, i.e., heme iron, are more readily bioavailable and absorbable in the small intestines compared to nonheme iron that is gotten from plant sources of iron such as green leafy vegetables. During routine antenatal clinic, pregnant women should be made aware of the important role of iron in pregnancy and development of the foetus as well as the different sources of iron and encouraged to consume more of the animal sources.

The mean WDDS reported in this study is higher than the 3.68 reported by Yeneabat et al. among pregnant women attending ANC in Ethiopia [21], 4.9 among pregnant women in Kenya [19], and 4.2 among pregnant women resident in rural areas in Northern Ghana [14]. It is however lower than the 6.84 reported by Kiboi et al. [12] among pregnant women from Laikipia county, Kenya. The differences could be due to seasonality, economic, and sociocultural differences.

In identifying the determinants of WDDS, we found that pregnant women who reportedly had higher income levels were more likely to have higher WDDS scores compared to their counterparts who had lower income levels. This finding is consistent with the findings of a study among a sample of pregnant women from Ethiopia, in which the authors found that pregnant women with “poorer” and “poor” wealth index had inadequate diet diversity [21], and those from Kenya [12] and India [28], in which inadequate dietary diversity was higher among those with lower income levels. A probable explanation could be the fact that higher income is associated with increased purchasing power and subsequently increased purchasing of different types of food and increased diet diversity. Interestingly, nutrition knowledge and attitudes were not associated with the dietary practices of pregnant women, consistent with the findings of previous studies reporting that nutritional practices are not associated with knowledge and attitudes but rather economic factors [16, 17, 29]. These findings demonstrate that future nutrition intervention projects need to not only concentrate on improving the nutrition knowledge and attitudes of pregnant women, but also include measures that could improve the income levels of pregnant women.

This study is not without limitations. Its cross-sectional nature makes it difficult to establish causality. Given that dietary diversity was assessed based on recall and self-reports, the findings may be liable to recall bias and social desirability bias. In addition, we also adapted a questionnaire that has been validated previously [4]. This is also an institution-based study, and the findings may not be generalizable. However, they provide a foundation for future studies to be built upon. Notwithstanding the limitations, the study has strengths worth noting. The findings increase our understanding of the dietary diversity of pregnant women in rural Ghana, which can serve as evidence to inform the design of future interventions to improve the dietary practices of pregnant women.

## 5. Conclusion

Dietary diversity of pregnant women was suboptimal. The consumption of vitamin A- and iron-rich food groups was inadequate. Income levels may be a more important determinant of dietary diversity of pregnant women than nutrition knowledge and attitudes. Although it is important to improve the nutrition knowledge and attitudes of pregnant women, future interventions should consider improving their income levels.

## Figures and Tables

**Table 1:** Sociodemographic characteristics of pregnant women.

Variable	Frequency (%)
*Marital status*	
Married/cohabiting/living together	122 (93.2)
Not married	10 (6.8)

*Occupation*	
Employed	122 (93.8)
Not employed	8 (6.2)

*Educational status*	
High	32 (24.6)
Low/no formal education	98 (75.4)

*Monthly income*	
≥GHC 500 ($87)	14 (10.1)
<GHC 500 ($87)	115 (88.9)

	Mean ± SD

Age	27.28 ± 6.68
Number of antenatal care visits	2.85 ± 1.99
Parity	2.36 ± 1.26
Gestation (in months)	6.24 ± 2.03
Household size	3.95 ± 1.87

$1 = GHC 5.78.

**Table 2:** Proportion of women consuming at least one food item from each of the food groups in the last 24 hours.

Food group	Frequency (%)
Starchy staples	130 (100.0)
Dark green leafy vegetables	122 (93.8)
Vitamin A-rich fruits, vegetables, and tubers	56 (53.1)
Other fruits and vegetables	111 (85.4)
Organ meats (iron rich)	10 (7.7)
Eggs	40 (30.8)
Meat and fish	112 (86.2)
Legumes, nuts, and seeds	81 (62.3)
Milk and milk products	23 (17.7)
Oils and fats	98 (75.4)
Presence of food taboos	3 (2.3)
Food cravings	41 (31.5)
Out-of-home eating	46 (35.4)
Mean ± SD women dietary diversity score	5.27 ± 1.35

Dietary diversity classification	
Low	9 (6.9)
Medium	72 (55.4)
High	49 (37.7)

**Table 3:** Women dietary diversity scores stratified by sociodemographic characteristics of the participants.

Variable	Mean ± SD	95% CI of mean	*p* value
*Occupation*			
Employed	5.31 ± 1.36	5.06–5.55	0.125
Unemployed	4.50 ± 0.55	3.93–5.07	

*Marital status*			
Married/cohabiting	5.30 ± 1.37	5.00–5.55	0.114
Single	4.75 ± 0.71	4.16–5.30	

*Educational level*			
High	6.13 ± 1.48	5.59–6.66	0.026
Low	4.99 ± 1.18	4.75–5.23	

*Monthly income*			
≥GHC 500 (US$ 86)	7.43 ± 1.02	6.84–8.02	<0.001
<GHC 500	5.01 ± 1.14	4.80–5.22	

*Food taboos*			
Yes	4.33 ± 1.16	1.46–7.20	0.224
No	5.29 ± 1.35	5.06–5.53	

*Food cravings*			
Yes	5.22 ± 1.74	4.67–5.77	0.776
No	5.29 ± 1.13	5.05–5.53	

*Outside home eating*			
Yes	5.57 ± 1.46	5.13–6.00	0.063
No	5.11 ± 1.26	4.83–5.38	

**Table 4:** Pearson correlation among parity, household size, age, gestational age, nutrition knowledge, attitude towards nutrition, and women dietary diversity scores.

Variable	Parity	HH	ANC visits	GA	DK	ATN	WDDS
Age	0.811^*∗∗*^	0.731^*∗∗*^	0.139	0.148	0.127	0.143	0.104
Parity	1	0.803^*∗∗*^	0.106	0.136	0.077	0.103	0.070
Household size (HH)			0.160	0.105	0.062	0.080	0.088
Antenatal visits (ANC)				0.800^*∗∗*^	0.431^*∗∗*^	0.195^*∗*^	0.304^*∗∗*^
Gestational age (GA)					0.323^*∗∗*^	0.240^*∗∗*^	0.135
Nutrition knowledge (NK)						0.317^*∗∗*^	0.395^*∗∗*^
Attitudes towards nutrition (ATN)							0.010

^*∗∗*^Correlation is significant at the 0.01 level (2-tailed). ^*∗*^Correlation is significant at the 0.05 level (2-tailed). WDDS: women dietary diversity score.

**Table 5:** Linear regression of determinants of women dietary diversity among pregnant women.

Variable	*B*	SE	95% CI	Partial correlation	*p* value
Age	−0.03	0.03	−0.08–0.03	−0.09	0.343
Employed	0.02	0.56	−1.09–1.12	0.00	0.975
Married	−0.48	0.28	−1.02–0.07	−0.16	0.895
Parity	0.15	0.18	−0.21–0.50	0.06	0.410
High level of education	0.25	0.30	−0.35–0.85	0.08	0.412
Earning ≥ GHC 500 (US$ 86)	1.82	0.46	0.90–2.73	0.35	<0.001
Household size	0.06	0.09	−0.13–0.24	0.06	0.552
Number of antenatal care visits	0.07	0.01	−0.13–0.26	0.06	0.494
Gestation	−0.04	0.08	−0.21–0.13	−0.04	0.663
Knowledge	0.08	0.09	−0.10–0.26	0.08	0.382
Attitude	−0.01	0.08	−0.17–0.15	−0.02	0.870
Food taboos	−0.62	0.68	−1.97–0.74	−0.08	0.369
Food cravings	−0.44	0.23	−0.89–0.21	−0.17	0.061
Out-of-home eating	0.33	0.22	−0.11–0.78	0.14	0.139

Adjusted *R*^2^ = 0.33; *F* = 5.58.

## Data Availability

The data used to support the findings of this study are available from the corresponding author upon request.
